# Efficacy and Neural Mechanisms of Eye Movement Desensitization and Reprocessing (EMDR) for Depression: A Systematic Review and Qualitative Synthesis

**DOI:** 10.31083/AP46465

**Published:** 2026-09-10

**Authors:** Ying Li, Jiali Liu, Xintong Wang, Guifang Chen

**Affiliations:** ^1^Department of Neurology, Affiliated Hospital of Zunyi Medical University, 563000 Zunyi, Guizhou, China; ^2^Department of Psychiatry, Affiliated Hospital of Zunyi Medical University, 563000 Zunyi, Guizhou, China; ^3^Key Laboratory of Brain Function and Brain Disease Prevention and Treatment of Guizhou Province, 563000 Zunyi, Guizhou, China

**Keywords:** Eye Movement Desensitization Reprocessing (EMDR), depression, neural mechanisms, systematic review, psychotherapy

## Abstract

**Background:**

Depression is a psychiatric disorder characterized by mood disturbances and cognitive impairment. Existing treatments, such as pharmacotherapy and cognitive-behavioral therapy (CBT), often present limitations, including delayed onset of efficacy and low adherence. Eye Movement Desensitization and Reprocessing (EMDR), a psychotherapy originally developed for post-traumatic stress disorder (PTSD), has shown potential in the treatment of depression.

**Methods:**

This systematic review synthesized evidence from 27 studies (14 randomized controlled trials, 10 single-arm studies, and three controlled studies) published between January 1990 and December 2024, integrating both behavioral outcomes and findings on neural mechanisms. Literature searches were conducted in PubMed, Web of Science, and ScienceDirect.

**Results:**

The findings indicate that EMDR demonstrates potential efficacy in alleviating depressive symptoms. The included studies involved diverse populations, including individuals with major depressive disorder, comorbid conditions, and trauma exposure, suggesting potential applicability across a range of clinical contexts. Preliminary neurobiological evidence has explored possible mechanisms involving modulation of prefrontal-limbic circuits and the integration of traumatic memories, providing initial insights into its therapeutic effects.

**Conclusions:**

Current evidence supports EMDR as a promising intervention for depression. However, further large-scale, high-quality studies are required to confirm its generalizability, identify optimal target populations, and clarify its underlying neurobiological mechanisms.

## Main Points

1. Innovation: This review attempts to provide the first systematic synthesis of evidence on the neural mechanisms underlying EMDR therapy for depression. We also explored its applicability across diverse patient populations, which may provide a preliminary reference framework for future research expanding the application of EMDR.

2. Rigorous Methodology: This review strictly adheres to the Preferred Reporting Items for Systematic Reviews and Meta-Analyses (PRISMA) guidelines. Qualitative analysis of 27 included studies indicates that EMDR therapy is associated with improvements in depressive symptoms across multiple clinical contexts.

3. Impact: The findings may inform the consideration of extending EMDR therapy to patients with non-traumatic depression and those with complex comorbidities; if confirmed by future research, this could help guide clinical practice and contribute information for developing individualized treatment pathways. However, the conclusions are constrained by significant heterogeneity and limitations within the evidence base.

## 1. Introduction

Eye Movement Desensitization and Reprocessing (EMDR) was developed by Francine Shapiro [1] in the late 1980s. It is a structured psychotherapy centered on facilitating the adaptive integration of traumatic memories through bilateral stimulation (e.g., eye movements). Its theoretical foundation is the Adaptive Information Processing (AIP) model, which posits that psychopathology arises from maladaptively stored, unprocessed traumatic memory [1]. EMDR aims to activate the brain’s innate information processing system to transform these memories into adaptive resolutions. Initially validated for post-traumatic stress disorder (PTSD), EMDR is now recognized as a first-line treatment for PTSD by organizations like the World Health Organization [2]. Its applications have expanded to include anxiety disorders [3], bipolar disorder [4], chronic dependency, and chronic pain [5].

Depression is a prevalent mental disorder and a leading cause of global disability [6]. Characterized by persistent sadness, anhedonia, and cognitive deficits, depression imposes a significant societal and economic burden [7]. Standard treatments, including antidepressant medications and cognitive behavioral therapy (CBT), have limitations such as side effects, variable response rates, and non-adherence [8]. A substantial subset of patients, particularly those with treatment-resistant depression (TRD) or comorbid conditions like trauma, do not achieve remission with first-line interventions [9]. Recent meta-analyses have summarized the growing evidence for EMDR in treating depression [10,11,12]. These reviews generally support EMDR’s efficacy, but the integration of neurobiological mechanisms remains limited. Understanding how EMDR affects the brain is crucial for validating its therapeutic mechanisms and optimizing its application.

Neurobiological models of depression implicate dysfunction within prefrontal-limbic circuits, including hyperactivity of the amygdala, reduced volume and function of the hippocampus, and diminished prefrontal regulation [13]. The AIP model suggests that EMDR may facilitate memory [1], which could in turn influence the neural circuits implicated in depression. Preliminary neuroimaging studies in PTSD and depression have reported EMDR-related changes in regions such as the anterior cingulate cortex (ACC) and prefrontal areas [14,15]. However, a systematic integration of this evidence specifically for depression is lacking.

This systematic review aims to: (1) update and qualitatively synthesize the current evidence on the clinical efficacy of EMDR for depressive disorders, including diverse populations (e.g., TRD, comorbid medical/psychiatric conditions); (2) systematically review and integrate preliminary findings on the neural mechanisms underlying EMDR’s effects on depression; and (3) identify limitations of the current evidence base and suggest directions for future research. By synthesizing clinical outcomes and preliminary neurobiological findings, this review aims to provide a balanced and critical overview examining the current evidence regarding EMDR’s potential role in treating depression, highlighting promising signals and significant knowledge gaps.

## 2. Materials and Methods

This systematic review was conducted and reported in accordance with the Preferred Reporting Items for Systematic Reviews and Meta-Analyses (PRISMA) 2020 guidelines [16]. Due to significant clinical and methodological heterogeneity observed among the included studies, a meta-analysis was deemed inappropriate; thus, a qualitative synthesis was performed.

### 2.1 Search Strategy

A systematic literature search was performed in three electronic databases: PubMed, Web of Science, and ScienceDirect, from January 1, 1990, to December 31, 2024. The search strategy combined terms related to EMDR and depression using Boolean operators. The whole search strategy for PubMed was as follows:

((“Eye Movement Desensitization Reprocessing” [Mesh] OR “EMDR” OR “Eye Movement Desensitization” OR “Bilateral stimulation”)) AND ((“Depression” [Mesh] OR “Depressive Disorder” [Mesh] OR “Depress” OR “Depressive Symptom” OR “MDD” OR “TRD”)).

Similar strategies were adapted for other databases (see **Supplementary Table 1**). Reference lists of included articles and relevant reviews were manually screened to identify additional studies.

### 2.2 Eligibility Criteria

Studies were included if they met the following criteria:

Population: Individuals with depressive symptoms, including major depressive disorder (MDD), TRD, or depressive symptoms comorbid with other conditions (e.g., PTSD, medical illnesses). All age groups were included.

Intervention: Standard EMDR therapy, including the core eye movement component. Studies using EMDR as a standalone intervention or as an adjunct to treatment-as-usual (TAU) were included, provided EMDR’s effects could be isolated.

Comparison: Any control condition (e.g., waitlist, TAU, active treatments like CBT) or no comparator (for single-arm studies).

Outcomes: The primary outcome was change in depressive symptom severity, measured by standardized scales (e.g., Beck Depression Inventory [BDI], Hamilton Depression Rating Scale [HAMD]). Secondary outcomes included neurobiological measures (e.g., Electroencephalography).

Study Design: Empirical studies including randomized controlled trials (RCTs), non-randomized controlled trials, cohort studies, and single-arm pre-post studies. Case reports, reviews, conference abstracts, and theoretical articles were excluded.

Only articles published in English in peer-reviewed journals were included.

### 2.3 Study Selection and Data Extraction

The study selection process followed the PRISMA flow diagram (Fig. 1). After duplicate removal, titles/abstracts were screened, followed by full-text assessment against eligibility criteria. Data were extracted using a standardized form that captured details on study design, population, intervention, outcomes, and results. Due to inconsistencies in effect size calculation methods (within-group vs. between-group) and the tendency for effect sizes in single-arm studies to be prone to overestimation, these data are not presented to avoid misleading interpretations. The extraction was performed independently by two reviewers (Cohen’s Kappa values consistently >0.85), with disagreements resolved through consensus or consultation with a third reviewer.

**Fig. 1.**
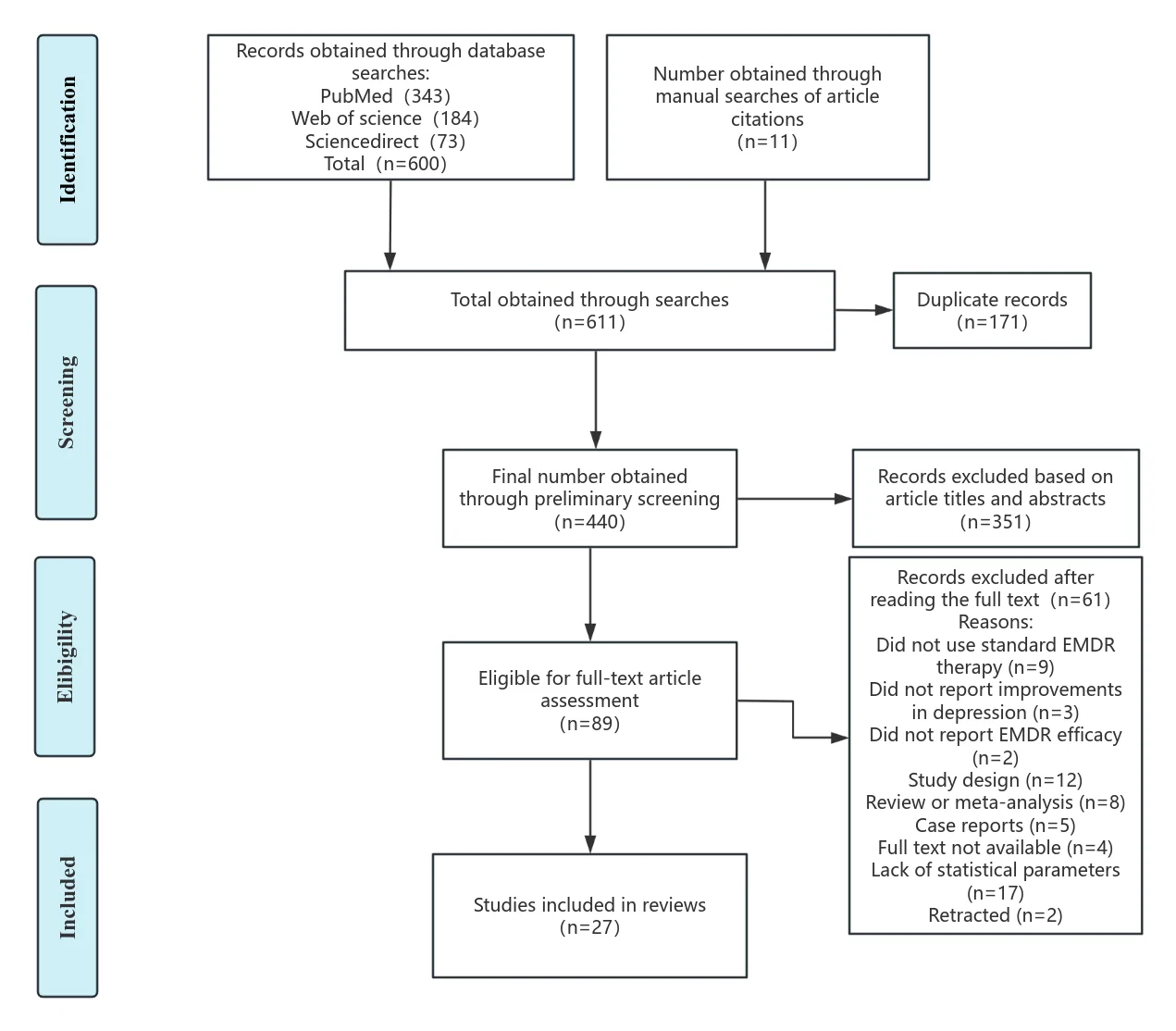
**PRISMA flowchart of study selection process**. PRISMA, Preferred Reporting Items for Systematic Reviews and Meta-Analyses.

### 2.4 Quality Assessment

Given that single-arm studies and non-randomized controlled trials cannot control for confounding factors in their design, they are unsuitable for inferring causal relationships between interventions and outcomes. Therefore, this study does not perform traditional risk-of-bias assessments on them but treats them as preliminary evidence. A formal risk of bias assessment was conducted only for RCTs. For the included RCTs, the Revised Cochrane risk-of-bias tool for randomized trials (RoB 2) [17] was used. Due to the expected difficulty of blinding therapists and participants in psychotherapy trials, the performance bias domain was excluded from the overall risk of bias judgment, as it would consistently be rated high and not discriminate between studies. Two reviewers independently conducted the assessments (Cohen’s Kappa values ranged from 0.78 to 1.00).

## 3. Results

### 3.1 Study Characteristics and Risk of Bias

The systematic search yielded 27 studies for inclusion (Fig. 1), comprising 14 RCTs, 10 single-arm pre-post studies, and 3 controlled non-randomized studies. The total sample size was approximately 1300 participants with depressive symptoms, spanning ages 7 to 70 years. Populations included individuals with MDD, TRD, depression comorbid with PTSD, and depression associated with somatic conditions (e.g., multiple sclerosis, myocardial infarction, tinnitus). Key study characteristics are detailed in Table 1 (Ref. [14,15,18,19,20,21,22,23,24,25,26,27,28,29,30,31,32,33,34,35,36,37,38,39,40,41]).

**Table 1.** **Detailed selection process of included studies**.

Sequence	Authors. year	Study design	Subgroup	Number of sample cases	Experimental group/control group	Age	Number of EMDR sessions(n)	Duration of each session (min)	Follow-up	Depression measurements	Primary diagnosis	Outcome
1	Meneses et al., 2024 [18]	RCT	EMDR vs. NET	105	51/54	34.30	10	60	—	HADS	Female victims of violence	The experimental group achieved greater reductions in anxiety, depression, and post-traumatic symptoms.
2	Zhao et al., 2023 [19]	RCT	EMDR vs. Waitlist	57	28/29	18–35	12	90	—	SDS	Clinical high-risk syndrome of PTSD comorbid with psychosis	Mean score reductions on all self-rating scales were greater in the EMDR group.
3	Meentken et al., 2021 [20]	RCT	EMDR vs. CAU	74	37/37	9.60 ± 2.90	3.53	50	2, 8 months	CDI-II	Subthreshold PTSD	Depression scales improved in both groups of children, but EMDR was not superior to CAU in reducing symptoms of PTSD, depression, and sleep problems.
4	Perri et al., 2021 [21]	RCT	EMDR vs. CBT	38	19/19	48.30 ± 13.60	7	—	1 month	BDI-II	Acute stress disorder	Depression scores decreased significantly in both groups; EMDR and CBT were equally effective, and both produced relevant improvements in outcome measures.
5	Rahimi et al., 2019 [36]	RCT	EMDR vs. TAU	90	45/45	51.52 ± 11.13	6	30–45	2 weeks	HADS	Hemodialysis population	Significantly lower levels of anxiety and depression in the EMDR group (post-treatment mean 6.27 vs. baseline 10.78, *p* < 0.05).
6	Jaberghaderi et al., 2019 [22]	RCT	CBT vs. EMDR	102	25/24/53	8–12	3–12	45–60	—	CROPS	Population with psychological sequelae in children	Both CBT and EMDR can help children recover significantly from the outcomes of domestic violence compared to the control group.
7	Hase et al., 2018 [23]	RCT	EMDR+TAU vs. TAU	30	14/16	40.32 (9.25)	8.50 (2.41)	—	—	BDI-II, SCL-90-R	MDD	BDI-II scores were significantly lower, and depressive symptoms were reduced more in the experimental group than in the control group.
8	Ostacoli et al., 2018 [41]	RCT	EMDR vs. CBT	66	31/35	48.23 (9.66)	15 ± 3	—	6 months	BDI-II	Depression	Depression scores decreased more significantly in the EMDR group than in the CBT group.
9	Acarturk et al., 2016 [24]	RCT	EMDR vs. Waitlist	70	37/33	33.32 (11.09)	4.20 (SD = 1.30)	—	1 month	BDI-II	PTSD	The results support the efficacy of EMDR in significantly reducing PTSD symptoms as well as depressive symptoms assessed with the BDI and HSCL.
10	Carletto et al., 2016 [25]	RCT.	EMDR vs. relaxation therapy	42	20/22	39.52 (11.68)	10	60	6 months	HADS	Multiple sclerosis, PTSD	Both treatments were effective in reducing the severity of PTSD, anxiety, and depressive symptoms and improving quality of life.
11	Acarturk et al., 2015 [26]	RCT	EMDR vs. Waitlist	29	15/14	19–63	4.13 (SD = 1.73)	90	1 month	BDI-II	PTSD	EMDR significantly reduces PTSD and depression symptoms.
12	Luyten et al., 2020 [27]	RCT	EMDR vs. CBT	89	46/43	47.87 (12.67)	5	60	3 months	HADS	Chronic subjective tinnitus	HADS was significantly lower in both groups, but there was no significant difference between groups.
13	Minelli et al., 2019 [28]	RCT	EMDR vs. TF-CBT	22	12/10	52.30	24 0	60	1, 2, 3, 6 months	BDI-II	Refractory depression	Depression improved in both groups, but depression scores were lower in the EMDR group at 3-month follow-up (*p* = 0.04), and efficacy persisted until 24 weeks.
14	Behnammoghadam et al., 2015 [29]	RCT	EMDR vs. Waitlist	60	30/30	50.97 ± 8.25	3	45–90	1 week	BDI	Myocardial infarction with depression	Depression significantly decreased in EMDR group (27.26 to 11.76); increased in control group (24.53 to 31.66); difference between groups *p* < 0.001.
15	Rovaris et al., 2024 [15]	Single-arm	EMDR	13	—	31.10 ± 8.90	12	—	3 months	HDRS	Multiple sclerosis with depressive symptoms	HDRS showed a significant reduction in depressive symptoms after treatment.
16	Baptist et al., 2021 [14]	Single-arm	EMDR	10	—	42.31 ± 15.03	10	75 ± 15	1 week	PHQ-9	Major depression	Significant decrease in PHQ-9 depression score.
17	Trentini et al., 2015 [30]	Single-arm	EMDR	10	—	9.53	8–10	—	—	TSCC-A	Children with complex trauma	Decreased scores on clinical scales of depression and post-traumatic stress.
18	Paauw et al., 2019 [31]	Single-arm	EMDR	25	—	15.80	6	60	3 months	CDI	Depression	Depressive symptoms (CDI: Cohen’s d = 0.72) were significantly reduced after treatment.
19	Rolling et al., 2024 [32]	Single-arm	EMDR	22	—	14.60	6	90	3 months	CDI	PTSD	Significant reduction in depression scores (CDI from 18.2 to 10.6).
20	Hamid et al., 2024 [33]	Single-arm	Online EMDR	83	—	33.20	<12	—	—	PHQ-9	PTSD	The proportion of depressive symptoms decreased from 95% at baseline to 16% at endpoint.
21	Brennstuhl et al., 2022 [34]	Single-arm	EMDR	21	—	45.10 (11.10)	4	60	1 week	HADS	COVID-19 hospitalized critically ill patients	EMDR Significant reduction in all symptoms (HADS depression score from 14.6 at baseline to 12.6 post-treatment).
22	Altmeyer et al., 2022 [35]	Single-arm	EMDR	49	—	46 (11.10)	12.88 (SD = 6.02)	—	3, 12 months	BDI-II	MDD	55% of patients were in complete remission at discharge (BDI-II <12); 74% of those in remission at 12 months had no recurrence.
23	Phillips et al., 2019 [36]	Single-arm	EMDR	14	—	57	9	60	6 months	BDI	Chronic tinnitus	Significant improvement in BDI score.
24	Wood et al., 2018 [37]	Single-arm	EMDR	13	—	46.00 ± 13.10	≤20	60	3 months	HRSD	Depression	Mean HRSD decreased by14.1 points.
25	Tang et al., 2015 [38]	Non-randomized control	EMDR vs. TAU	83	41/42	14.20 ± 0.99	8–32	30–40	—	CES-D	Adolescents with significant mental disorders	Significant improvement in self-reported depression through EMDR intervention.
26	Hofmann et al., 2014 [39]	Non-randomized controlled	EMDR+TAU vs. TAU	42	21/21	40.38 (10.38)	7	—	—	BDI-II	Depression	Patients in the EMDR combined with CBT group had a more pronounced decrease in BDI-II scores.
27	Hase et al., 2015 [40]	Matched-pair design	EMDR+TAU vs. TAU	32	16/16	46.41 (9.06)	4.60 (SD = 2.40)	—	12–16 months	SCL-90-R	Depression	The EMDR group showed a greater reduction in depressive symptoms.

Note: —, indicates data not reported (or not available); EMDR, Eye Movement Desensitization and Reprocessing; RCT, randomized controlled trial; PTSD, post-traumatic stress disorder; CBT, cognitive-behavioral therapy; TF-CBT, trauma-focused cognitive-behavioral therapy; MDD, major depressive disorder; HADS, Hospital Anxiety and Depression Scale; SDS, Self-Rating Depression Scale; TAU, treatment as usual; CDI-II, Children’s Depression Inventory-2; BDI-II, Beck Depression Inventory-II; SCL-90-R, Symptom Checklist-90-Revised; MS, multiple sclerosis; MI, myocardial infarction; HDRS, Hamilton Depression Rating Scale; PHQ-9, Patient Health Questionnaire-9; TSCC-A, Trauma Symptom Checklist for Children-Alternate Version; COVID-19, Coronavirus Disease 2019; HRSD, Hamilton Rating Scale for Depression; CES-D, Center for Epidemiologic Studies Depression Scale; NET, Narrative Exposure Therapy; CROPS, Child Report of Post-traumatic Symptoms; CAU, care-as-usual.

Risk of bias assessment of the 14 RCTs using RoB 2 showed a predominance of low-risk ratings across several domains, particularly for random sequence generation, blinding of outcome assessment, and selective reporting (Fig. 2). However, the risk of bias due to deviations from intended interventions and the selection of the reported result was often unclear.

**Fig. 2.**
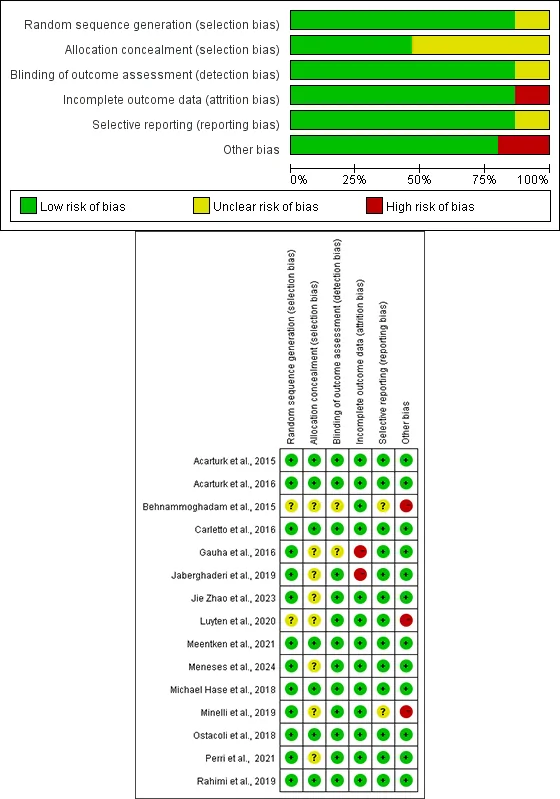
**Risk of Bias Summary**. Green plus sign (+) = low risk of bias; red minus sign (−) = high risk of bias; yellow question mark (?) = unclear risk of bias.

### 3.2 Clinical Efficacy Outcomes

The included studies suggest that EMDR may yield clinical benefits across diverse populations and treatment settings. However, its efficacy was not necessarily superior to other existing therapies. Results from direct comparative studies were inconsistent, with some reports indicating EMDR may offer greater advantages, while others found it to be comparable to control therapies.

#### 3.2.1 EMDR as a Standalone or Adjunctive Intervention

Multiple RCTs have demonstrated that EMDR effectively reduces depressive symptoms compared to waitlist controls [24,26,29], and in specific contexts, its efficacy is comparable to or superior to TAU [23]. As an adjunctive treatment, EMDR combined with TAU showed greater symptom reduction than TAU alone in several studies [39,40]. Single-arm studies reinforced these findings, showing significant pre-post reductions in depression scores [14,35,37].

#### 3.2.2 Comparisons With Active Treatments (e.g., CBT)

Findings regarding comparisons with CBT were mixed. Some RCTs found EMDR to be superior to TF-CBT in reducing depression scores among inpatients with TRD at follow-up [28] and to CBT in patients with recurrent depression [41]. However, other studies found no significant differences in depressive symptom reduction between EMDR and CBT [21,27]. One study in children with subthreshold PTSD found both EMDR and CAU improved depression, but EMDR was not superior [20].

#### 3.2.3 Specific Populations

EMDR showed beneficial effects in specific groups:

Children and Adolescents: Significant reductions in depressive symptoms were reported [30,31,38], with some studies noting sustained effects [20].

Comorbid Medical Conditions: EMDR was associated with improved depression in patients with multiple sclerosis [15,25], myocardial infarction [29], tinnitus [27,36], and those undergoing hemodialysis [42].

Trauma-Exposed Populations: Refugees [24,26], victims of violence [18,22], and individuals with comorbid PTSD [19] showed significant improvements in depressive symptoms post-EMDR.

### 3.3 Neurobiological Findings

Three studies (one RCT nested imaging study, two single-arm neuroimaging studies) investigated neural correlates of EMDR in depressed samples Table 2 (Ref. [14,15,30]). Sample sizes were small (n = 10 to 13).

**Table 2.** **Details of imaging studies included in the study**.

Authors and year	Neuroimaging technique	Core metrics	Key findings	Study population	Experimental methods	Supported hypotheses on underlying mechanisms
Rovaris et al., 2024 [15]	fMRI (task-based)	Brain activation in different emotional conditions (positive vs. neutral, adverse vs. neutral)	Enhanced activation in the left premotor area and reduced activation in the right inferior parietal lobule.	Patients with MS with depression	MRI scans were performed before and after EMDR.	Corrected negative cognitive bias
Baptist et al., 2021 [14]	EEG	Frontal theta cordance (fTC/pfTC)	Symptom improvement correlated with reduced frontal theta cordance.	Adults with monophasic depression	A 2-minute resting EEG (eyes closed) was recorded at the beginning of each EMDR.	Restoration of prefrontal-limbic system function
Trentini et al., 2015 [30]	hdEEG	Source localization activation strength	Shift in brain activation from the right prefrontal-limbic system to left temporal lobe regions.	Children with complex trauma	Recorded once before and after treatment—100–400 ms post-stimulation (event-related potentials).	Promoting traumatic memory integration

Note: fMRI, functional magnetic resonance imaging; EEG, Electroencephalography; TC, theta cordance; fTC, frontal lobe theta cordance; pfTC, prefrontal lobe theta cordance; MS, multiple sclerosis; hdEEG, high-density EEG.

Baptist et al. (2021) [14] found that clinical improvement following EMDR in adults with MDD was correlated with a reduction in frontal theta cordance (fTC) at F3/F4/Fz sites.

Rovaris et al. (2024) [15] used task-fMRI in patients with multiple sclerosis and depression, observing that EMDR-induced symptom improvement was associated with decreased activation in the right parietal lobe and increased activation in the left premotor area during negative emotional processing.

Trentini et al. (2015) [30] reported a shift from right prefrontal-limbic hyperactivity to increased left temporal activation in traumatized children after EMDR, consistent with enhanced memory integration per the AIP model.

## 4. Discussion

The 27 studies ultimately included exhibited high design heterogeneity. Therefore, we refrained from quantitative synthesis and instead conducted an in-depth qualitative analysis based on the available evidence. This review systematically examines the efficacy profile, scope of effects, and potential mechanisms of EMDR in depression intervention across multiple dimensions. While several meta-analyses have established EMDR’s efficacy for depression [10,11,12], this review extends the literature by systematically incorporating emerging neurobiological evidence and discussing its clinical implications.

### 4.1 Discussion of Behavioral Outcomes

This study synthesizes 14 randomized controlled trials, 10 single-arm studies, and three controlled studies. Despite diverse methodological designs, the findings reveal both encouraging trends and substantial heterogeneity: across the included studies, EMDR was frequently associated with reduced depressive symptoms across various populations. Its effects appear particularly pronounced in individuals with traumatic experiences [21,27,28,41]. However, examining the evidence base necessitates acknowledging its high internal variability. For instance, among studies targeting children, Jaberghaderi et al. (2019) [22] compared EMDR with CBT, while Meentken et al. (2021) [20] used standard care as the control. Naturally yielding differing conclusions [20,22]. This inherent heterogeneity in study design precludes the establishment of a uniform effect size. It also demands more refined stratification to determine for whom and under what conditions EMDR may be most effective.

#### 4.1.1 Efficacy of Standalone vs. Combined Application

As a standalone intervention, some studies suggest that EMDR may lead to symptom reduction within a relatively small number of sessions. For instance, in patients with treatment-resistant depression, study reports indicate that symptoms show significant reduction after an average of 3.5–4 treatment sessions [28]. Some studies also suggest that its improvement effects may be comparable to or slightly different from CBT in certain contexts. One study found greater reductions in BDI-II scores in the EMDR group compared to the CBT group [41]. Minelli et al. (2019) [28] observed more stable long-term outcomes in treatment-resistant depression patients receiving EMDR during follow-up.

However, existing evidence is not entirely consistent. Some studies failed to identify a clear advantage of EMDR over CBT. For instance, no statistically significant difference was observed between EMDR and CBT for alleviating depressive symptoms in patients with chronic tinnitus [27]. These conflicting findings highlight the complexity of the current evidence, partly due to the variability in what constitutes ‘CBT’ across different RCTs. Perri et al. (2021) [21] applied it to acute stress disorder, while Ostacoli et al. (2018) [41] used it for depression, resulting in inherently different protocols and emphases. This renders “EMDR vs. CBT” comparisons, to some extent, a comparison against a non-fixed control rather than a strictly consistent direct contrast.

When combined with standard care, some studies support EMDR’s potential additive effect. Adding EMDR to conventional treatment may be associated with greater reductions in depressive symptoms [39,40]. For patients with suboptimal response to traditional approaches, EMDR may serve as a complementary option. However, broader incorporation into current treatment guidelines likely requires further well-designed, clearly comparative clinical trials.

#### 4.1.2 Population-Specific Efficacy

Beyond treatment format, the efficacy of EMDR also appears to vary across different age groups, though its value manifests differently. For minors, its value lies in circumventing the high demands of traditional therapies on abstract thinking and verbal expression [43]. For instance, a study involving children with an average age of 9.6 years reported reduced medical trauma-related fears and depressive symptoms after an average of 3.5 treatment sessions [20]. Its shorter treatment duration may enhance compliance; another study demonstrated a 60.9% remission rate for adolescent depression [31]. Similarly, depressive symptoms in the adult population were also alleviated [33]. Some authors suggest that the structured, less verbally demanding format of EMDR may align well with the developmental characteristics of minors, who may have less developed abstract thinking and emotional expression skills [43].

EMDR has also been studied in adult populations with limited access to or response to traditional mental health interventions. Several studies report positive outcomes for refugees [24,26], severe COVID-19 survivors [34], and myocardial infarction patients [29]. These groups are termed “underserved” due to their high stress levels and often limited access to existing psychological interventions. However, it is important to note that current studies targeting these populations are generally small-scale. For instance, the sample size for COVID-19 patients was 21, and for multiple sclerosis patients, it was 13 [15,34].

#### 4.1.3 Efficacy for Comorbid and Non-Comorbid Depression

The studies included in this review demonstrate that EMDR yields positive therapeutic effects for various patient groups with comorbid depression, though the strength and specificity of the evidence vary across populations and control settings [19,25,27,42]. In comparisons between active treatment and passive controls (such as waiting lists or routine care), EMDR has shown superiority [19,29,42]. However, when directly compared with other structured active psychotherapies (e.g., cognitive behavioral therapy or relaxation therapy), the relative advantage of EMDR is less clear [25,27]. These findings suggest that EMDR may be an effective intervention for comorbid depressive symptoms, but its efficacy may not be unique and could be comparable to other evidence-based therapies in certain contexts [25,27]. The heterogeneity of current evidence (encompassing diverse populations, including those at psychotic risk and pediatric complex trauma) suggests broad potential applicability [19,30], yet further high-quality controlled studies are needed to clarify its efficacy and scope.

Regarding EMDR’s efficacy for non-comorbid depression, current evidence presents a positive yet preliminary picture [23,28,40]. Higher-level evidence—randomized controlled trials comparing EMDR to standard care or active treatments like CBT—indicates EMDR effectively reduces depression scores and may yield more enduring benefits [23,28]. Concurrently, multiple single-arm studies provide supporting data for these effects [31,37]. These findings challenge the common perception that “EMDR is primarily applicable to trauma-related disorders”. However, critical evaluations of this evidence base indicate its overall strength is limited. First, some key conclusions rely on studies with small sample sizes. Second, certain positive findings originate from single-arm studies without controls, whose conclusions require validation through more rigorous designs.

#### 4.1.4 Treatment Parameters and Long-Term Efficacy

The optimal dosage for EMDR remains to be fully investigated. Table 1 reveals significant variations in treatment parameters, ranging from three ultra-short sessions for post-myocardial infarction depression [29] to 24 intensive sessions for treatment-resistant depression [28]. Overall, shorter interventions (≤ 4 sessions) demonstrate higher efficacy for well-defined stress-related issues, while chronic or complex cases typically require longer treatment courses. This dosage variation also reflects current methodological limitations in research, namely the lack of systematic exploration of dose-response relationships. For instance, why did an average of 3.53 sessions [20]. yield limited effects on depressive symptoms in children with subthreshold PTSD, whereas an average of 3 sessions [29] significantly improved post-myocardial infarction depression? These discrepancies may relate to population characteristics, target issues, or therapeutic focus. Few studies provide detailed analyses of treatment completion rates or response timelines, making it challenging to determine the minimum effective dose and optimal treatment duration across different clinical contexts.

Regarding treatment durability, existing evidence generally shows positive trends. Multiple studies indicate effects can be sustained for 3 to 6 months [28,31,37], while Altmeyer et al. (2022) [35] reported continued positive outcomes at the 12-month follow-up. However, it should be noted that long-term follow-up studies spanning a full year remain scarce. Moreover, this study employed a single-arm design, lacking a control group to account for factors such as the natural course of the disorder. Therefore, the long-term efficacy of EMDR in preventing relapse requires further validation through additional high-quality research.

#### 4.1.5 Expanded Efficacy in Implementation Settings

EMDR therapy demonstrates a degree of flexibility in its implementation. The emergence of online EMDR has expanded the scope of this therapy in terms of service accessibility [33]. While emerging online formats may enhance accessibility, their efficacy and safety compared to in-person delivery remain understudied. Additionally, research has explored the possibility of EMDR being delivered by non-psychiatric professionals (e.g., nurses) [42], which provides a practical foundation for its larger-scale application within public health systems. A short-term intervention study in a post-disaster school setting showed an association between psychologist-led EMDR intervention and a reduction in depressive symptoms among adolescents after a disaster [38], suggesting the therapy may have potential applicability in non-typical clinical settings.

Meanwhile, this flexibility is accompanied by several issues that remain to be clarified. When EMDR moves from more strictly controlled research environments to complex real-world situations or is administered by non-specialists who have received only brief training, concerns arise regarding the consistency of the treatment process. Whether the effects of EMDR delivered by providers with different professional backgrounds are comparable has not been sufficiently concluded by existing research [33,38,42]. Therefore, in the process of expanding the use of EMDR, how to establish corresponding standards and oversight mechanisms to maintain its effectiveness across different contexts has become a noteworthy issue.

### 4.2 Discussion of Neurological Mechanisms Results

EMDR was initially developed for PTSD [44]. Its mechanism of action in PTSD provides critical theoretical support for extending its application to other disease domains, particularly depression. However, studies specifically examining the antidepressant mechanisms of EMDR remain limited [14,15,30], highlighting the necessity and urgency of systematically reviewing and integrating existing evidence. The research underlying this section is extremely limited in quantity and sample size, so its findings are entirely preliminary and interpretive in nature, rather than establishing definitive mechanisms.

#### 4.2.1 Restoration of Prefrontal-Limbic System Function

An EEG study demonstrated that improvements in depressive symptoms correlate with reduced prefrontal theta synchrony (at F3/F4/Fz electrodes) [14]. This shift in neural activity is interpreted as reflecting normalization of prefrontal cortical function, leading to the hypothesis that EMDR may alleviate depressive symptoms by regulating neural activity in this region [14]. Synthesizing these findings, EMDR’s antidepressant effects are thought to potentially involve restoring prefrontal-limbic system function. Similar mechanisms have been reported in PTSD research: A SPECT brain imaging study demonstrated enhanced prefrontal cortical activity and reduced temporal association cortex activity following EMDR treatment, which is thought to help correct functional imbalances between the limbic system and prefrontal cortex [45]. Collectively, these findings suggest that regulating functional connectivity between the prefrontal cortex and limbic systems may represent one potential pathway through which EMDR exerts its therapeutic effects.

#### 4.2.2 Correcting Negative Cognitive Biases

Depression often manifests as a neural cycle pattern characterized by a cascade: amygdala overactivation leads to insufficient prefrontal regulation, which in turn enhances rumination and ultimately suppresses reward function” [46,47]. This pattern is considered the neural basis for negative cognitive bias. Research has explored whether EMDR produces therapeutic effects by altering this pathological circuit. A Task-based fMRI study found that following EMDR treatment, patients exhibited reduced activation in the right inferior parietal lobule and increased activation in the left premotor area during negative information processing. These changes in neural activity were temporally synchronized with improvements in depressive symptoms [15]. A theoretical model proposed that EMDR’s two parallel processes, namely attentional control enhancement and bilateral stimulation, act together to reduce inhibition, thereby enabling dysfunctional emotional information to be processed [48]. Based on these findings, which indicate that EMDR may promote emotional processing through dual pathways, “enhancing emotional release” and “reducing excessive inhibition”.

#### 4.2.3 Facilitating Traumatic Memory Integration

Alternatively, EMDR’s efficacy in improving depressive symptoms may be related to its promotion of traumatic memory integration. Studies indicate that memory integration deficits positively correlate with depression severity [49,50]. Clinical observations reveal that patients undergoing EMDR therapy exhibit integration, improved working memory and other cognitive functions alongside reduced depressive symptoms [51]. Neuroimaging research provides further observational evidence supporting this association. One fMRI study demonstrated a shift in brain activation from a right prefrontal-limbic system-dominant pattern to left temporal lobe activation following EMDR treatment [30]. Another study documented a similar trend, with brain activity shifting from limbic emotional processing regions to temporal-occipital cognitive integration areas [52]. This transition is interpreted as a shift of traumatic memories from an emotion-driven state to a cognitively integrated state, consistent with the “adaptive information processing” model proposed in EMDR theory [53]. Collectively, these findings suggest that facilitating the integration of traumatic memories may represent a potential mechanism through which EMDR alleviates depressive symptoms.

### 4.3 Limitations of the Evidence Base

Although this review strictly followed the PRISMA guidelines, its conclusions remain constrained by multiple limitations in the existing evidence and should be interpreted with caution. First, studies exhibit high heterogeneity with significant variations in design, populations, and intervention parameters, complicating quantitative synthesis. Second, sample sizes are generally small and limited to English-language literature, potentially affecting statistical power and generalizability while introducing language bias. Third, blinding is challenging to implement, and due to the nature of EMDR therapy, studies commonly carry high risks of implementation and measurement bias. Fourth, inconsistent control group designs, particularly when comparing EMDR to “active treatments” like cognitive behavioral therapy—lack standardized control interventions, undermining comparability. Fifth, neurobiological evidence remains weak, with limited studies and small samples yielding mostly preliminary inferences. Finally, long-term efficacy and real-world application data are scarce, lacking extended follow-up. The effectiveness and standardization of EMDR in non-specialized settings and complex clinical contexts remain unvalidated.

### 4.4 Future Directions

Future research should focus on deepening exploration in the following areas. The primary task is to conduct large-sample, long-term follow-up randomized controlled trials to confirm its sustained efficacy and potential for relapse prevention. Second, specialized neuroimaging studies targeting depressed populations should be conducted. Multimodal techniques should be employed to validate its neural mechanisms directly. Concurrently, systematic exploration of the relationship between treatment dosage and response is needed to develop optimal protocols for different patients. Furthermore, scientific research should be strengthened to evaluate its scalability across diverse settings (e.g., online interventions). Finally, integrating clinical outcomes with biomarkers will advance EMDR toward personalized applications.

## 5. Conclusions

Existing research indicates that EMDR shows potential in alleviating depressive symptoms, with its efficacy surpassing waiting lists or conventional treatments in multiple studies. However, results remain inconsistent when directly compared with active interventions such as cognitive behavioral therapy, and its relative advantages remain unestablished. Current investigations span diverse populations, including children and adolescents, individuals with comorbid conditions, and trauma-exposed individuals, suggesting broad clinical applicability. Preliminary evidence regarding neural mechanisms suggests its effects may involve regulating prefrontal-limbic system function and information processing patterns. However, the number of relevant studies is limited, and conclusions remain speculative. The current evidence base is constrained by small sample sizes and high heterogeneity. Further high-quality clinical and translational research is essential to firmly establish EMDR’s efficacy, clarify its mechanisms of action, and define its optimal role within comprehensive depression treatment strategies.

## Data Availability

The data extracted from the included studies for this systematic review are available from the corresponding author upon reasonable request.
