# Supplementary material for: Efficacy and Neural Mechanisms of Eye Movement Desensitization and Reprocessing (EMDR) for Depression: A Systematic Review and Qualitative Synthesis
Source: Alpha Psychiatry. 2026 Sep 10;27(5):46465. doi: 10.31083/AP46465 (PMC13731988; doi:10.31083/AP46465)
Supplement: Supplementary file 1 [file 2757-8038-27-5-46465-s1.zip › Supplementary Table 1.docx]

Supplementary Material

| Databases | Search formula |
| --- | --- |
| PubMed  (n = 343) | **#1 ("Eye Movement Desensitization Reprocessing" OR EMDR OR "Bilateral stimulation")**  **#2 ("Depression"[Mesh] OR "Depressive Disorder"[Mesh] OR "Depress*" OR "Depressive Symptom*" OR "Emotional Depression" OR "MDD" OR "TRD")**  **#1 AND #2** |
| **Web of Science**  (**n = 184**) | **All Fields = (("Eye Movement Desensitization Reprocessing" OR EMDR OR "Bilateral stimulation")**  **AND**  **("Depress*" OR "Depressive Disorder" OR "MDD" OR "TRD*" OR "dysthymia"))** |
| ScienceDirect  (n = 73) | TITLE-ABSTR-KEY = (("Eye movement desensitization reprocessing" **OR EMDR** OR **"Bilateral stimulation"**)  AND  ("depression" OR "depressive disorder" OR "MDD" OR "TRD" OR **"dysthymia"**)) |

Note:search formats used in various databases.
